# Lineage-specific Virulence Determinants of *Haemophilus influenzae* Biogroup aegyptius

**DOI:** 10.3201/eid1803.110728

**Published:** 2012-03

**Authors:** Fiona R. Strouts, Peter Power, Nicholas J. Croucher, Nicola Corton, Andries van Tonder, Michael A. Quail, Paul R. Langford, Michael J. Hudson, Julian Parkhill, J. Simon Kroll, Stephen D. Bentley

**Affiliations:** Imperial College London, London, UK (F.R. Strouts, P.R. Langford, J.S. Kroll);; University of Oxford, Oxford, UK (P. Power);; Wellcome Trust Sanger Institute, Cambridge, UK (N.J. Croucher, N. Corton, A. van Tonder, M.A. Quail, J. Parkhill, S.D. Bentley);; The Health Protection Agency, Salisbury, UK (M.J. Hudson)

**Keywords:** Haemophilus influenzae, Brazilian purpuric fever, BPF, Haemophilus influenzae biogroup aegyptius, Haemophilus aegyptius, trimeric autotransporter, trimeric autotransporter adhesins, adhesin, sepsis, pathogenicity, bacteria, virulence

## Abstract

An emergent clone of *Haemophilus influenzae* biogroup aegyptius (*Hae*) is responsible for outbreaks of Brazilian purpuric fever (BPF). First recorded in Brazil in 1984, the so-called BPF clone of *Hae* caused a fulminant disease that started with conjunctivitis but developed into septicemic shock; mortality rates were as high as 70%. To identify virulence determinants, we conducted a pan-genomic analysis. Sequencing of the genomes of the BPF clone strain F3031 and a noninvasive conjunctivitis strain, F3047, and comparison of these sequences with 5 other complete *H. influenzae* genomes showed that >77% of the F3031 genome is shared among all *H. influenzae* strains. Delineation of the *Hae* accessory genome enabled characterization of 163 predicted protein-coding genes; identified differences in established autotransporter adhesins; and revealed a suite of novel adhesins unique to *Hae*, including novel trimeric autotransporter adhesins and 4 new fimbrial operons. These novel adhesins might play a critical role in host–pathogen interactions.

## Abstract 2

Novel adhesions, including trimeric autotransporters, might contribute to virulence.

For more than a century, *Haemophilus influenzae* biogroup aegyptius (*Hae*) has caused worldwide seasonal epidemics of acute, purulent conjunctivitis (*1**,**2*). In 1984, an entirely new syndrome, Brazilian purpuric fever (BPF), emerged in the town of Promissão, São Paulo State, Brazil. Caused by an emergent clone of *Hae*, the virulence of BPF in children was unprecedented and fatal. Invasive infection was preceded by purulent conjunctivitis that resolved before the onset of an acute bacteremic illness, which rapidly evolved into septic shock complicated by purpura fulminans (*3*). In the 11 years to 1995, several hundred cases of BPF were reported, of which all but 3 were in Brazil (*4**,**5*); overall mortality rate was 40%. Cases occurred sporadically and in outbreaks, mainly in small towns, although some were in the state capital, where an epidemic was feared because of crowding and deprivation. A collaborative task force by the Brazilian Health Authorities and the US Centers for Disease Control and Prevention was created to investigate this emergent infection and identified the cause as the BPF clone of *Hae* (*Hae*BPF) (*6*).

After 1995, no more cases were reported for more than a decade, although cases may have been missed, submerged in periodic surges of clinically indistinguishable hyperendemic or epidemic meningococcal disease. The potential of the disease to reappear with devastating effect is, however, underscored by the recent report of a suspected outbreak (7 cases, 5 fatal within 24 hours) in 2007 in the town of Anajás in the previously unaffected Brazilian Amazon region (*7*); thus, it cannot be assumed that this emergent infection has gone away.

The emergence of new pathogens causing human and animal diseases represents a constant threat. Distinguishing invasive strains from their noninvasive relatives is relevant for diagnosis, treatment, and prevention of the spread of emerging infectious diseases. *Hae*BPF constitutes a unique *H. influenzae* clade separate from the usual conjunctivitis-causing *Hae* strains (*8*); in experimental infections, it has caused sustained septicemia (*9*) and endothelial cytotoxicity (*10*).

However, despite intensive research spanning 2 decades, these phenotypes remain unexplained. *Hae*BPF, a strain of nontypeable *H. influenzae* (NTHI), lacks genes encoding the polysaccharide capsule, a major virulence determinant of invasive *H. influenzae.* Although 1 animal study has indicated that a phase-variable lipopolysaccharide structure might play a part in the serum resistance of *Hae*BPF (*11*), in other respects, a novel lipopolysaccharide has not convincingly explained its virulence (*12*). With regard to adhesins, Farley et al. (*13*) identified duplication of fimbrial (*haf*) genes, with sequences differing from *H. influenzae* type b pilin (*hif*), but could find no systematic difference in binding of *Hae*BPF and conventional *Hae* strains to human epithelial cells and could not conclusively implicate this locus in virulence. Various other BPF-specific outer membrane proteins potentially involved in host–pathogen interactions have been identified, including a partially characterized hemagglutinin (*14*) and an ≈145-kDa phase-variable protein eliciting protective immunity (*15*), but none have been fully characterized, and their role in disease has not been established. *Hae*BPF (but not other *Hae* strains) has a copy of the *Haemophilus* insertion element IS*1016* (*16*), which has been implicated in acquisition of capsulation genes and other unspecified virulence factors in other *H. influenzae* strains (*17**,**18*), but its role has not been defined.

To better define the role of *Hae*BPF, we conducted a pan-genomic analysis. This comparison with 5 other complete *H. influenzae* genomes available in public databases has enabled delineation of the accessory genome for *Hae* and *Hae*BPF, characterizing all *Hae*-specific features that might contribute to the differences in the biology of this lineage of *H. influenzae*. This study goes beyond other *H. influenzae* pan-genome studies (*19*) by comparing only complete genomes and provides an absolute genomic comparison among the strains. Analysis of differences in genome content between the *Hae* strains and other *H. influenzae* revealed a plethora of novel adhesins that might play a critical role in host–pathogen interactions.

## Materials and Methods

We first sequenced and annotated the genomes of the *Hae*BPF strain F3031 and a contemporaneous, non–BPF-associated conjunctivitis strain from Brazil, F3047. We compared strains F3031 and F3047 with *H. influenzae* strain Rd KW20, the type d capsule-deficient laboratory strain that was the first free-living organism to have its genome sequence determined; with *H. influenzae* strain 10810, a serotype b meningitis strain; with NTHI strains 86–028NP and R2846 (strain 12) (*20*), isolated from middle ear secretions from patients with otitis media; and with NTHI strain R2866, an unusually virulent NTHI strain isolated from a child with meningitis.

### Bacterial Strains Sequenced

F3031 (GenBank accession no. FQ670178) is a BPF clone strain that is indistinguishable from other isolates by various typing systems, including multilocus sequence typing. F3047 (GenBank accession no. FQ670204) is a conjunctivitis isolate from Brazil that was established by typing to be unrelated to the BPF clone. F3031 and F3047 are described in more detail elsewhere (*21*).

### Sequencing and Assembly

Bacterial genomes were sequenced at the Wellcome Trust Sanger Institute, Cambridge, UK. The first drafts of the F3031 and F3047 genomes were assembled from sequence to ≈7-fold coverage, from pOTWI2 and pMAQ1Sac_BstXI genomic shotgun libraries, by using BigDye Terminator chemistry on an Applied Biosystems 3730 DNA Analyzer (Applied Biosystems, Foster City, CA, USA). End sequences from large insert fosmid libraries in pCC1FOS (insert size 38–42 kb) were used as a scaffold for each strain. Further sequencing was performed on the Illumina Genome Analyzer (Illumina, Inc., San Diego, CA, USA). Assemblies were created and gaps and repeat regions were bridged by read pairs and end-sequenced PCR products.

### Annotation and Analysis

Coding sequences were predicted by using Glimmer 3 (www.cbcb.umd.edu/software/glimmer). Automated annotation by similarity was done by searching the Glimmer 3 coding sequence set against the National Center for Biotechnology Information Clusters of Orthologous Groups database and the SwissProt dataset (www.uniprot.org). Annotation by similarity was done by importing the NTHI strain 86–028NP annotation and comparing it with the F3031 coding sequence set by using reciprocal FASTA (www.ebi.ac.uk/Tools/sss/fasta). Automated annotation was confirmed by manual curation with the Artemis genome visualization tool (*22*). Gene definitions and functional classes were added manually by using FASTA analyses of the primary automated comparisons. tRNA genes were predicted by using tRNAScan-SE version 1.2 (*23*). Identification of the rRNA operons was based on similarity to homologs in the NTHI strain 86–028NP genome.

### Pan-Genome Comparison

Generation of pairwise comparisons of complete genome sequences was based on alignment of basepairs in MAUVE (*24*), which enabled alignment of whole genome sequences despite rearrangements. For each pairwise comparison of whole-genome sequences, the length of the alignment between the 2 strains was calculated and a distance matrix was created. The distance matrix, based on the lengths of the sequence alignments, was used to create a heat map showing the clustering of strains.

### Phylogenetic Analysis

Evolutionary relationships between protein-coding sequences from different strains were inferred by using MEGA version 5.02 (*25*). Phylogenetic trees were constructed by using sequence alignments, and a neighbor-joining tree was built under a Poisson correction substitution model assuming uniform rates of substitutions among sites.

## Results

### The 7 Complete *H. influenzae* Genomes

Genome sizes ranged from 1.83 to 2.0 Mb (Table 1). The F3031 genome comprises 1,985,832 bp, is 8% larger than Rd KW20, and encodes 1,892 genes. The F3047 genome is larger (2,007,018 bp) and encodes 1,896 genes. All strains have a genome G+C content of 38%, typical of *H. influenzae*. *Hae*BPF strain F3031 contains an ≈24-MDa plasmid, previously sequenced and annotated (*26*), with average G+C content of 36.7%. This plasmid has been excluded from analysis.

**Table 1:** *Haemophilus influenzae* strains included in pan-genome comparison*

*S*train	Disease	Serotype	Genome size, Mb	G+C content, %	Identified CDSs	Sequencing location
F3031	Brazilian purpuric fever	Nontypeable	1.99	38.2	1,892	WTSI, Imperial College
F3047	Conjunctivitis	Nontypeable	2.0	38.2	1,896	WTSI, Imperial College
Rd KW20	Laboratory strain	d, capsule-deficient	1.83	38.1	1,743	JCVI
86–028NP	Otitis media	Nontypeable	1.91	38.2	1,821	Ohio State University
10810	Meningitis	b	1.98	38.0	1,896	WTSI, Oxford University
R2846	Otitis media	Nontypeable	1.98	37.0	1,691	University of Washington, SBRI
R2866	Meningitis	Nontypeable	1.89	38.0	1,817	University of Washington, SBRI

*CDSs, coding sequences; WTSI, Wellcome Trust Sanger Institute; JCVI, J. Craig Venter Institute; SBRI, Seattle Biomedical Research Institute.

**Figure 1:**
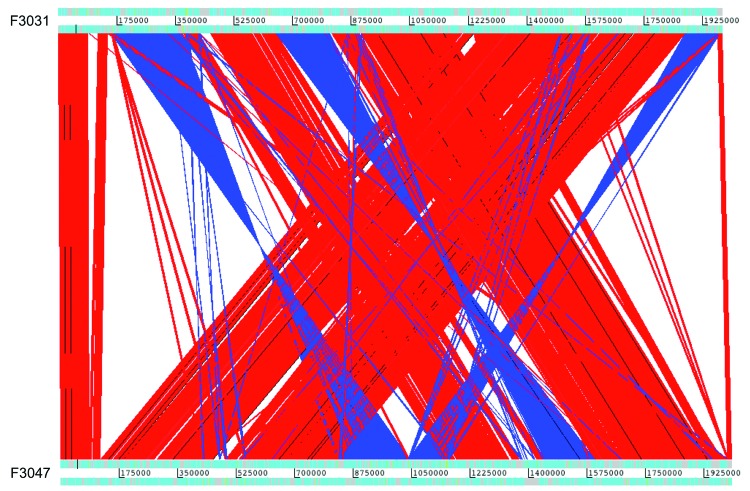
Comparison of the whole genome of Brazilian purpuric fever clone of *Haemophilus influenzae* biogroup aegyptius (*Hae*BPF) strain F3031 and *Hae* conjunctivitis strain F3047 with Artemis Comparison Tool (*22*). Red, syntenic regions; blue, inverted regions of the genome.

**Figure 2:**
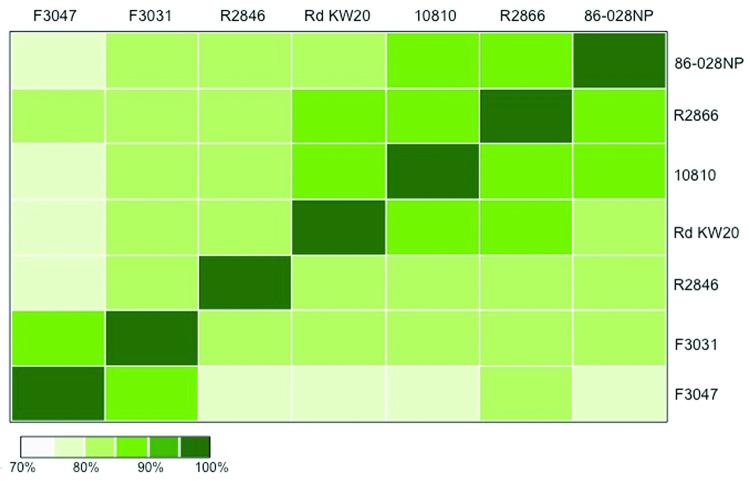
Pair-wise comparisons of genome alignments between 7 *Haemophilus influenzae* strains. Each colored block represents the total number of bases shared between 2 *H. influenzae* genomes. Scale bar indicates percent relatedness.

Whole-genome alignment of *Hae* strains F3031 and F3047 revealed substantial colinearity with 1 major rearrangement and 3 small inversions (Figure 1). Pairwise nucleotide alignments of the 7 sequences indicated a closer relatedness of the 2 *Hae* strains to each other than to the 5 other *H. influenzae* genomes (Figure 2). A core genome of 77% was shared across all 7 strains.

### The *Hae* Accessory Genome

**Figure 3:**
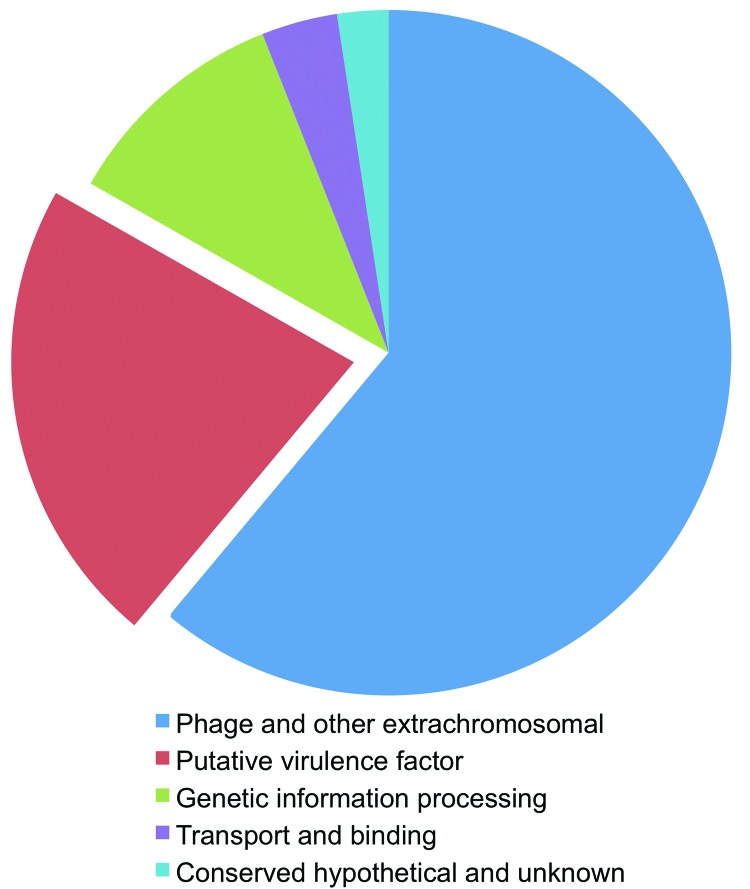
*Haemophilus influenzae* biogroup aegyptius (*Hae*)–specific features (163 coding sequences [CDSs]) determined from the pan-genome comparison. Putative virulence factors (red) accounted for ≈22% (13 CDSs) of all features identified.

F3031 shares 10.6% of its genomic sequence with 1 other strain and 88% of this shared sequence (9.3% of total) with F3047, emphasizing the closer relatedness of these 2 strains to each other than to the other *H. influenzae* strains. A total of 163 predicted coding sequences lie within this *Hae*-specific DNA. A total of 99 (61%) coding sequences lie within regions of previously characterized *Haemophilus* bacteriophages, encoding proteins inferred by the similarity of their deduced sequences to be phage components associated with coexpressed genes transported by the phage (phage cargo). These proteins are are either homologs of conserved hypothetical proteins in other organisms or previously unidentified proteins of unknown function. Of all *Hae*-specific genes, >22% encoded homologs of products identified elsewhere as being involved in host–pathogen interactions; prominent members were putative adhesins and invasins not previously found in strains of *H. influenzae* (Figure 3). Description of the *Hae* accessory genome will focus on these putative adhesins.

These new *Hae*-specific adhesins include 4 novel fimbrial operons, unique high-molecular-weight (HMW) proteins, and a 10-member family of trimeric autotransporter adhesins (TAAs). Many of these coding sequences are associated with simple sequence repeats (SSRs), indicating that phase variation may confer the potential for antigenic variation and immune response evasion during infection.

**Figure 4:**
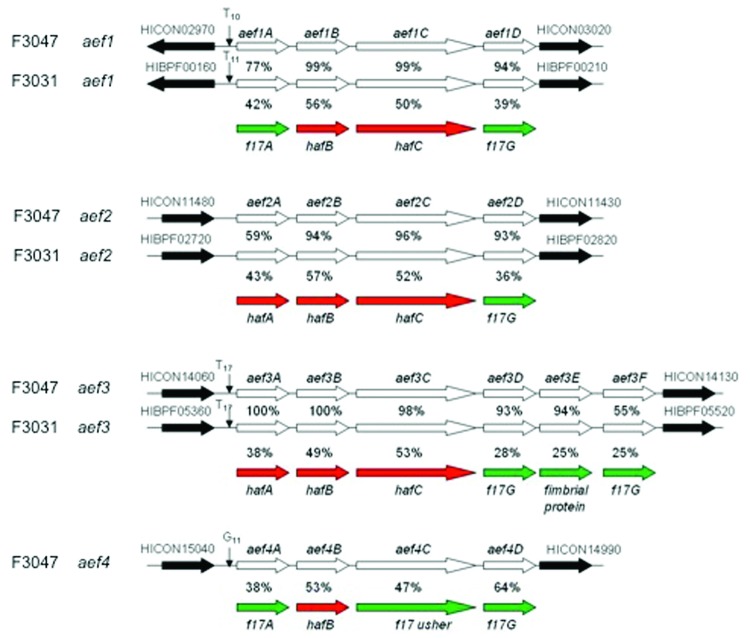
*aef* fimbrial operons in *Haemophilus influenzae* biogroup aegyptius strains F3047 (1–4) and F3031 (1–3). The *aef* fimbrial genes in each putative cluster are indicated by open arrows, and the flanking genes by solid arrows. The genes encode *aefA* (a fimbrial protein), *aefB* (a fimbrial chaperone), *aefC* (a fimbrial usher protein), *aefD* (a fimbrial adhesion), and *aef3E* and *aef3F* (additional fimbrial adhesins). Simple sequence repeats in the promoter region for each gene cluster are shown. Percent sequence identity between the aef genes from F3047 and F3031 is given between respective genes. Percent identity to closest homologue in Hae (red arrows) or other organisms (green arrows) is shown by features below each operon. BPF, Brazilian purpuric fever; CON, conjunctivitis.

The presence of duplicated *hafABCDE* operons (*27*) was confirmed in the F3031 and F3047 genomes. We also identified 4 more *Hae*-specific fimbrial gene clusters, *aef1*–*aef4* (Figure 4). Clusters *aef1–aef3* were present in both strains, although not identical (55%–100% similarity on gene-by-gene comparison), but *aef4* was not present in *Hae*BPF F3031. Each *aef* operon encodes 4–6 proteins and has modest sequence identity to products of corresponding *haf* genes (38%–57%) and to F17 fimbrial adhesins (25%–64%) produced by pathogenic *Escherichia coli* associated with septicemic diarrheal diseases. Three clusters (*aef1*, *aef3*, *aef4*) are associated with mononucleotide SSRs of 10–17 nt located in the putative promoter region upstream of the *aefA* gene (Figure 4), conferring capacity for phase-variable expression through expansion and contraction of the SSR, altering efficiency of promoter binding.

**Figure 5:**
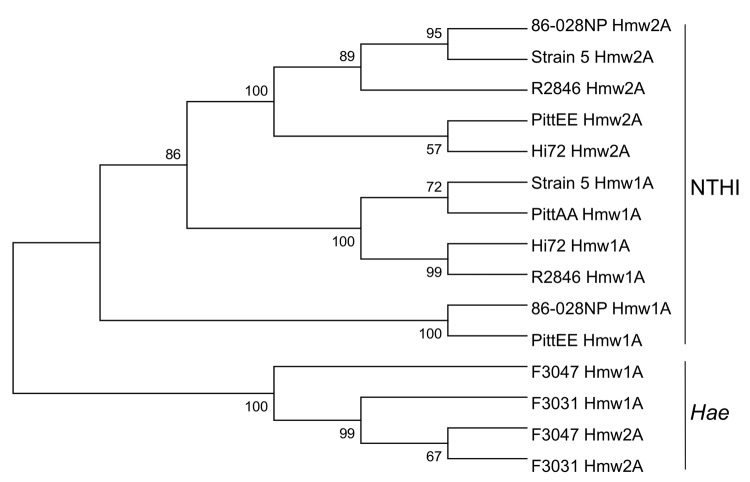
Phylogenetic relatedness of HmwA binding domain. Neighbor-joining tree based on the predicted binding domain of the HmwA adhesins from the indicated nontypeable *Haemophilus influenzae* (NTHI) strains, constructed by using MEGA5.02 (*25*). Bootstrap confidence values are shown at the branches, based on 1,000 replications. The population divides into 2 major clusters; HmwA alleles from nontypeable *H. influenzae* strains are clearly separated from *H. influenzae* biogroup aegyptius (*Hae*).

The *Hae* genomes each encode a much richer repertoire of autotransporter adhesins than is found in other sequenced *Haemophilus* spp. Monomeric (classical) and novel trimeric autotransporter adhesins are present (MAA and TAA, respectively). Of the established *Haemophilus* autotransporter adhesins, the MAA Hap (*Haemophilus* adhesion and penetration protein), widely distributed in *H. influenzae* and proposed as a candidate NTHI vaccine antigen, is present as a pseudogene in F3031 and F3047, as previously reported by Kilian et al. (*28*). IgA1 protease, previously identified in the BPF clone, is also present in conjunctivitis strain F3047. Sequence alignment to other *H. influenzae* demonstrated that IgA1 from F3047 is more closely related to IgA1 from Rd KW20 (88% aa identity) than from F3031 (65% aa identity). Homologs of the HMW adhesins HMW1 and HMW2 (MAAs) and of the TAA *H. influenzae* adhesin Hia are present in F3031 and F3047. In contrast to the many NTHI strains for which substantial sequence information is available, where HMW1/HMW2 or Hia have almost always been alternatives, both are found in these *Hae* strains. HMW1 and HMW2, encoded at loci each consisting of 3 genes (*hmwABC*), were first identified in NTHI strain R2846 as HMW surface-exposed proteins, mediating attachment to human epithelial cells (*29*). More than 75% of NTHI encode HMW1 and HMW2, present at the same chromosomal locations in almost all HMW-containing NTHI isolates examined. *hmw1A* and *hmw2A* encode adhesins with different receptor binding specificities resulting from domains in variable regions comprising amino acid residues 114–237 of mature Hmw1A and 112–236 of mature Hmw2A (*30*). Despite conservation in binding specificity, *hmw1A* or *hmw2A* alleles from different isolates are highly polymorphic in the receptor binding domains (*30*). *Hae* Hmw1A– and Hmw2A–binding domain sequences (deduced from comparison with R2846 sequence) were aligned by using ClustalW (www.ebi.ac.uk/Tools/msa/clustalw2) with those from homologs in other NTHIs, regardless whether they were Hmw1A or Hmw2A, and the alignment was used to construct a phylogenetic tree (Figure 5). The *Hae* HmwA–binding domains are distinct from those in other NTHIs, suggesting that in *Hae* these proteins have diverged separately from other NTHIs.

In *Hae* F3031 and F3047, the HMW clusters are not at the homologous NTHI chromosomal position; they are elsewhere, with a 22-kb bacteriophage insertion directly downstream of *hmw2ABC*. The *hmwA* alleles are further differentiated from those found in other NTHI strains by their associated SSRs. The putative promoter region upstream of the *hmw1A* and *hmw2A* homologs contains the octanucleotide repeat unit 5′-GCATCATC-3′; there are 14 and 15 copies, respectively, in F3031 and 13 and 12 copies, respectively, in F3047. This repeat pattern contrasts with all *hmwA* genes so far sequenced in different NTHI strains, in which 7 basepair SSRs of either 5′-ATCTTTC-3′ or 5′-TGAAAGA-3′ in varying copy numbers are located upstream of the genes (*31**,**32*).

The *Hae* accessory genome includes a 10-member gene family that encodes proteins with the sequence characteristics of TAAs (Table 2). These TAAs are distinct from the *Haemophilus* TAAs Hsf (*33*), Hia (*34*), or the recently described Cha (*35*). In strains F3031 and F3047, a total of 8 genes (*1**–**6**,**8**,**9*) are present as homologs, termed *tabA* (for the *Hae*BPF trimeric autotransporter [bpf] alleles) or *tahA* (for the regular *hae* [conjunctivitis] alleles). *tahA7* has no homlog in *Hae*BPF. The tenth gene, *tabA10*, is the recently described adhesin/invasin gene *hadA* (*36*). This gene is found only in *Hae*BPF F3031; F3047 has no corresponding gene. Each gene appears to be locus specific, sharing the same flanking regions, but sequences differ substantially between homologs 1 and 2 in particular. *tabA4/tahA4, tabA5* and *tabA9/tahA9* seem to be pseudogenes, carrying frameshift mutations within the coding sequence. All TAAs except *tabA8/tahA8* and *tabA10* (*hadA*) are associated with SSRs located either within the coding sequence or upstream in the putative promoter region, indicating that expression may be modulated by phase variation.

**Table 2:** New trimeric autotransporter adhesin proteins identified from genome sequences of *Haemophilus influenzae* biogroup aegyptius strains F3031 and F3047*

Name	Protein	Total length, aa	Molecular weight, kDa	G+C content, %	SSR, promoter CDS/CDS^−^
TabA1	HIBPF06240	1,490	140	47	TA (8) pr
TahA1	HICON14840	1,182	119	43	TA (5) pr
TabA2	HIBPF05270	2,185	211	47	G (13) CDS
TahA2	HICON14020	2,233	206	48	G (20) CDS
TabA3	HIBPF07130	464	41	38	CAAA (14) CDS^–^
TahA3	HICON16690	464	42	39	CAAA (12) CDS^–^
TabA4†	HIBPF07270	857	88	40	T (12) pr
TahA4†	HICON16820	759	77	41	T (10) pr
TabA5†	HIBPF10940	847	88	42	CAAA (15) pr
TahA5	HICON05410	1,016	106	41	CAAA (30) pr
TabA6	HIBPF01360	484	50	41	GCAA (16) CDS^–^
TahA6	HICON03690	484	50	41	GCAA (24) CDS
TahA7	HICON13720	905	95	39	GCAA (23) CDS^–^
TabA8	HIBPF01360	260	28	42	GCAA (3) CDS^–^
TahA8	HICON03690	282	30	43	GCAA (19) CDS^–^
TabA9†	HIBPF08080	232	25	36	NA
TahA9†	HICON17550	232	25	36	NA
TabA10 (HadA)	HIBPF19140	256	27	35	NA

*SSR, simple sequence repeats. Numbers in parentheses indicate the number of mono/di/tetranucluotide repeats found in each SSR; pr, SSR located within predicted promoter (pr) region upstream of the coding sequence (CDS); CDS, SSR located within the CDS; CDS^–^, SSR in CDS results in a frame-shift and an out-of-frame CDS; NA, not applicable. †Pseudogene.

**Figure 6:**
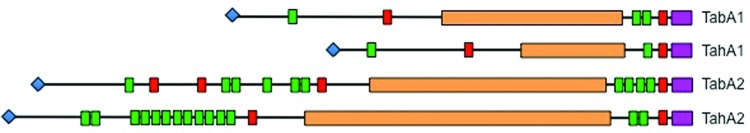
Domain organization of the *Haemophilus influenzae* biogroup aegyptius trimeric autotransporter adhesins TabA1/TahA1 and TabA2/TahA2, showing differences in passenger domain sequence motifs. Purple, C-terminal translocator domain; red, hemagglutinin domains; green, Hap_Hag domains; orange, degenerate repeats; blue, N terminal signal peptide.

All these TAAs share the characteristic 3-domain structure of N-terminal signal peptide and C-terminal outer membrane translocator domain, separated by an internal passenger domain. However, comparison of orthologous TAAs revealed striking differences between their passenger domains for TabA1/TahA1 and TabA2/TahA2, suggesting different functions of these proteins in the 2 strains (Figure 6). The passenger domains of these proteins vary in the number of binding domains (hemagglutinin and Hep_Hag domains) and in possession of different-sized, low-complexity spacer regions consisting of approximate heptapeptide repeats. TabA1 from F3031 contains 90 copies of tandemly duplicated AASSSAS with occasional T, N, or other substitutions in many copies; TahA1 from F3047 contains 48 copies of tandemly duplicated AETAKAG with occasional R, V, or other substitutions in many copies. In the prototypic TAA YadA, a series of 15-residue repeats appears to have such a spacer function between the protein head and its anchor in the outer membrane (*37*), holding any receptor-binding domains away from the bacterial cell surface.

**Figure 7:**
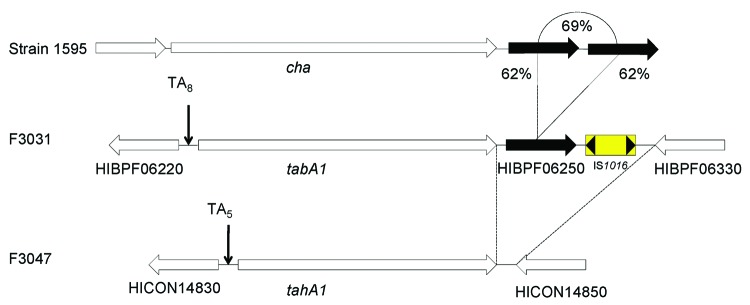
Comparison of the *cha* locus from *Haemophilus* cryptic genospecies strain 1595 to the TabA1 locus in the Brazilian purpuric fever (BPF) clone of *H. influenzae* biogroup aegyptius (*Hae*BPF) F3031 and the TahA1 locus in *H. influenzae* biogroup aegyptius (*Hae*) conjunctivitis (CON) F3047. Strain F3031 includes an additional 2 coding sequences downstream of *tabA1*, HIBPF06250 and IS*1016,* that are absent from strain F3047. HIBPF06250 is a conserved hypothetical protein with homology (62% aa identity) to the 2 coding sequences located directly downstream of *cha* that share 69% aa identity with each other.

In the context of the unusual virulence of the *Hae*BPF clone, the *tabA1* locus is particularly intriguing. Comparison with the *tahA1* locus indicates not only the substantial difference between the genes themselves, in the sequence encoding the putative stalk domain, but also (in F3031) an additional gene, HIBPF06250, encoding a conserved hypothetical protein, homologous to an uncharacterized gene product in the *Haemophilus* cryptic genospecies strain 1595 (*35*). In this strain, the gene (tandem duplicated) lies downstream of the TAA Cha. In F3031, HIBPF06250 is interposed between *tabA1* and IS*1016* (Figure 7), and the gene (like the insertion sequence) is absent in F3047. Association of IS*1016*, first described as the *Haemophilus* capsulation locus–associated insertion sequence, with unusual and invasive virulence of NTHI strains has been suggested elsewhere (*17**,**18*), although no specific gene association has been identified.

### The *Hae*BPF-specific Accessory Genome

The part of the *Hae* accessory genome unique to *Hae*BPF amounted to 102,304 bases (5.2% of its genome). Ten *Hae*BPF-specific loci ranged in size from 370 to 20,002 bases and in G+C content from 27.9% to 44.5%. Deviation from the *Haemophilus* average of 38% suggests that these are more recently acquired regions. Much of this DNA is located within 5 bacteriophage domains, containing all 219 coding sequences (12 *Hae* specific, 11 *Hae*BPF specific) (Table 3) and including 1 (phage region 1) now termed HP3, similar in size and gene content to *Haemophilus* bacteriophage HP2, found in NTHI strains associated with unusual virulence (*38*). The *Hae*BPF-specific accessory genome comprises these and another 10 coding sequences (Table 4), which remained apparently BPF specific after BLASTP analysis of their deduced amino acid sequences against the nondegenerate public databases (October 2011), which include many more *Haemophilus* sequences from incomplete genome sequencing projects (*19*). The nearest matches to these sequences were mainly homologs in other pathogenic bacterial species that occupy the same ecologic niche. One gene (*hadA* at BPF-specific locus 10) has recently been characterized as encoding an epithelial adhesin/invasin plausibly contributing to *Hae*BPF virulence (*36*), but the function of the others, and any part their products may play in the serum resistance of the *Hae*BPF clone that endows it with pathogenic potential, remains to be established. Eleven genes appear to be phage cargo (Table 3); these are either homologs of conserved hypothetical proteins identified in other organisms or entirely unknown and might represent novel virulence factors. Four F3031-specific gene products do not have homologs in any other bacterial species and cannot be assigned a putative function. Novel genes have generally formed a much larger part of newly sequenced bacterial genomes, and identification of so few unknown genes in *Hae*BPF strain F3031 reflects the current availability of a large amount of *Haemophilus* sequence data, in particular from strains of NTHI.

**Table 3:** Phage loci identified in genome of Brazilian purpuric fever clone of *Haemophilus influenzae* biogroup aegyptius strain F3031*

Phage region	Cluster start	Size, kb	G+C content, %	No. genes	No. *Hae-*specific (*Hae*BPF-specific) genes	Closest phage/gene product homologs
1	85,874	32	40.5	35	5 (5)	*Haemophilus* bacteriophage HP1, HP2, S2
2	325,263	47	41.2	60	0 (0)	Putative phage-related proteins from *H. influenzae, Neisseria meningitidis*
3	418,932	33	40.0	38	2 (2)	Mu-like phage from *H. influenzae*, *Mannheimia haemolytica*
4	857,914	54	39.5	60	5 (3)	Putative phage-related proteins from *H. influenzae, N. meningitidis*
5	1,240,967	30	40.6	26	0 (1)	Mu-like phage from *H. influenzae, H. somnus, H. parasuis*

*Hae, Haemophilus influenzae biogroup aegyptius; BPF, Brazilian purpuric fever.

**Table 4:** Coding sequences specific to *Haemophilus influenzae* biogroup aegyptius strain F3031 at 10 loci*

Locus no. and F3031 ID	G+C, %	% Identity	Predicted product	Species harboring closest homologue
1, phage region 1†				
HIBPF00881	41.2	60	Conserved hypothetical protein	*Neisseria meningitidis*
HIBPF00900	37.6	68	Plasmid maintenance system killer	*Haemophilus parasuis*
HIBPF00910	40.2	82	Plasmid maintenance system antidote protein	*Neisseria gonorrhoeae*
HIBPF01110	40.2	70	Conserved hypothetical protein	*H. parasuis*
HIBPF01260	38.5	NA	Unknown protein, no known homologs	NA
2, phage region 3†				
HIBPF04833	40.1	NA	Unknown protein, no known homologs	NA
HIBPF04834	37.6	54	Conserved hypothetical protein	*H. parasuis*
3				
HIBPF05360	27.5	NA	Unknown protein, no known homologs	NA
4, phage region 4†				
HIBPF09220	41.2	55	Conserved hypothetical protein	*Haemophilus haemolyticus*
HIBPF09642	31.2	NA	Unknown protein, no known homologs	NA
HIBPF09722	31.9	80	Conserved hypothetical protein	*H. parasuis*
5, phage region 5†				
HIBPF13250	44.1	75	Conserved hypothetical protein	*H. parasuis*
6				
HIBPF16620	37.5	65	Adenine-specific methyltransferase (pseudo)	*Mannheimia haemolytica*
HIBPF16630	38.1	71	HNH endonuclease	*M. haemolytica*
7				
HIBPF17711	32.6	53	Conserved hypothetical protein	*Escherichia coli*
HIBPF17712	26.5	49	Conserved hypothetical protein	*N. meningitidis*
8				
HIBPF18000	28.1	52	DNA methyltransferase	*Macrococcus caseolyticus*
HIBPF18010	26.7	56	DNA methyltransferase	*M. caseolyticus*
HIBPF18040	30.3	44	Restriction endonuclease	*M. caseolyticus*
9				
HIBPF19140	35.3	100	HadA trimeric autotransporter adhesin	Previously identified in *Hae*BPF
HIBPF20030	36.1	77	Antibiotic biosynthesis monooxygenase	*Aggregatibacter aphrophilus*

*Putative product based on closest homologue in public databases, shown by percent amino acid identity. Percentage G+C content given for each coding sequence. ID, identification; NA, not applicable. †Genes occur within regions of bacteriophage.

## Discussion

Although the unique virulence of the BPF clone of *Hae* might result from its acquisition of few (or even just 1) novel gene(s), our analysis indicates that sequence variation and variable gene expression through phase variation plausibly play a major role. Among the 21 *Hae*BPF-specific genes, just 1, *hadA* (*36*), is readily identifiable as a determinant of pathogenic behavior (virulence). This, however, is but 1 member of a new family of *Haemophilus* TAAs, which is unique to *Hae* but (except for *hadA*) shared among conjunctivitis isolates (12 diverse strains probed, unpub. data, the authors) and among members of the BPF clonal lineage (4 examples probed, unpub. data, the authors). Striking differences in sequences within the passenger domains of homologous TAAs indicate the possibility of differences in function, perhaps loss of epithelial localization through alteration of >1 of these adhesins in the *Hae*BPF clone. The abundance of other genes encoding putative adhesins, which differentiates *Hae* from other *H. influenzae*, underscores the early observation (*39*) that pilus and nonpilus factors mediate interactions of *Hae* with human cells in vitro. An understanding of expression of these multiple adherence factors will probably provide insights into *Hae* pathogenesis.

Comparison of complete, rather than draft or partially assembled, sequences leads to hypothesis-generating insights, which enable inferences as to possible gene function and clarification of phenotypic observations made before genomic information became available. For example, the pathogen-specific ≈145-kDa phase-variable protein identified by Rubin (*15*) can now be identified with some confidence as the intriguing TAA TabA1 (1 of few *Hae*BPF proteins predicted to be of this size and phase variable as a result of the SSR in the promoter region), enabling future investigations of its role in BPF virulence. The set of iron-regulated proteins identified experimentally by Smoot et al. (*40*) also should be identifiable by using a bioinformatic approach, greatly facilitating future study of this phenotype.

The next challenge is to experimentally test such hypotheses. Functional studies in *Hae*BPF have been hampered by the difficulty of genetically manipulating these strains, a difficulty that genomics does not explain. In silico analysis demonstrated that strains of *Hae* appear to encode all genes and regulatory sites needed for *H. influenzae* competence and transformation. Although small amino acid substitutions are found in most of the proteins when compared with homologs in readily transformable Rd KW20, not enough is known about their individual functions to enable prediction as to whether particular residue changes might affect function.

Our *H. influenzae* pan-genomic analysis demonstrated a close relationship between the *Hae*BPF strain F3031 and the conjunctivitis strain F3047. This finding contrasts with the remote relationship suggested by previous phylogenetic analyses (*8*). Analyzing complete genomes overcomes the limited discriminatory power of typing methods like multilocus sequence typing and, in this instance, supports the proposition that *Hae* strains are closely related and have a gene content that partially reflects their mucosal niche specificity.

The growing number of complete bacterial genomes provides increasing potential for comprehensive pan-genomic comparisons of related strains that vary in pathogenic potential. Such comparisons might reveal strain-specific features involved in virulence, which could lead to development of genotyping methods for tracking emerging pathogens and of new vaccines. Comparison of *Hae* with other strains of *H. influenzae* has detected novel candidate virulence determinants (the families of TAAs and fimbrial adhesions) that plausibly confer selective advantages in adapting to upper respiratory tract and conjunctival mucosae. It is tempting to speculate that alteration through mutation in the specificity of adhesins such as the TAAs might, as with *Hae*BPF, have created a maladaptive phenotype less firmly localized to the mucosal surface and able to invade the bloodstream. To investigate the role that the novel family of TAAs might play in host–pathogen interactions, we are conducting in vitro studies of gene function.
